# “Positive Regulation of RNA Metabolic Process” Ontology Group Highly Regulated in Porcine Oocytes Matured* In Vitro*: A Microarray Approach

**DOI:** 10.1155/2018/2863068

**Published:** 2018-01-10

**Authors:** Piotr Celichowski, Mariusz J. Nawrocki, Marta Dyszkiewicz-Konwińska, Maurycy Jankowski, Joanna Budna, Artur Bryja, Wiesława Kranc, Sylwia Borys, Sandra Knap, Sylwia Ciesiółka, Michal Jeseta, Karolina Piasecka-Stryczyńska, Ronza Khozmi, Dorota Bukowska, Paweł Antosik, Klaus P. Brüssow, Małgorzata Bruska, Michał Nowicki, Maciej Zabel, Bartosz Kempisty

**Affiliations:** ^1^Department of Histology and Embryology, Poznan University of Medical Sciences, Swiecickiego 6 St., 60-781 Poznan, Poland; ^2^Department of Anatomy, Poznan University of Medical Sciences, Swiecickiego 6 St., 60-781 Poznan, Poland; ^3^Department of Biomaterials and Experimental Dentistry, Poznan University of Medical Sciences, Bukowska 70 St., 60-812 Poznan, Poland; ^4^Department of Obstetrics and Gynecology, University Hospital and Masaryk University, Obilni trh 11, 602 00 Brno, Czech Republic; ^5^Veterinary Center, Nicolaus Copernicus University in Torun, Gagarina 11 St., 87-100 Torun, Poland; ^6^Department of Histology and Embryology, Wroclaw University of Medical Sciences, 6a Chalubinskiego St., 50-368 Wroclaw, Poland

## Abstract

The cumulus-oocyte complexes (COCs) growth and development during folliculogenesis and oogenesis are accompanied by changes involving synthesis and accumulation of large amount of RNA and proteins. In this study, the transcriptomic profile of genes involved in “oocytes RNA synthesis” in relation to* in vitro* maturation in pigs was investigated for the first time. The RNA was isolated from oocytes before and after* in vitro* maturation (IVM). Interactions between differentially expressed genes/proteins belonging to “positive regulation of RNA metabolic process” ontology group were investigated by STRING10 software. Using microarray assays, we found expression of 12258 porcine transcripts. Genes with fold change higher than |2| and with corrected *p* value lower than 0.05 were considered as differentially expressed. The ontology group “positive regulation of RNA metabolic process” involved differential expression of AR, INHBA, WWTR1, FOS, MEF2C, VEGFA, IKZF2, IHH, RORA, MAP3K1, NFAT5, SMARCA1, EGR1, EGR2, MITF, SMAD4, APP, and NR5A1 transcripts. Since all of the presented genes were downregulated after IVM, we suggested that they might be significantly involved in regulation of RNA synthesis before reaching oocyte MII stage. Higher expression of “RNA metabolic process” related genes before IVM indicated that they might be recognized as important markers and specific “transcriptomic fingerprint” of RNA template accumulation and storage for further porcine embryos growth and development.

## 1. Introduction

The mammalian oocytes grow and develop during long stages of folliculogenesis and oogenesis, which are substantially regulated and orchestrated by expression of clusters of target genes. The final step of female gamete growth following successful monospermic fertilization is oocyte maturation that takes place during oviduct transport. The oocytes may undergo maturation both* in vivo* and/or* in vitro*, but this process always involves significant morphological and molecular changes within the cells [1]. The maturation is divided into nuclear, which is characterized by chromosome reorganization that finally leads to reaching MII stage, and cytoplasmic, which includes a storage of RNA and proteins in cytoplasm. The RNA, which is accumulated within the oocyte cytoplasm, displays a significant function as the template for protein synthesis after fertilization and during embryo preimplantation development [2, 3]. Moreover, the oocyte's proteins catalyzed biochemical processes crucial for further embryo growth such as amino acid and fatty acid metabolism and/or early embryo morphogenesis. It was clearly demonstrated that the total amount of mRNA, which is stored and accumulated during cumulus-oocyte complexes (COCs) maturation, may be recognized as the main marker of successful oocyte growth [4, 5].

In this study, we aimed to investigate the transcriptome profile of porcine oocyte before and after 44 hours of* in vitro* maturation (IVM). “Positive regulation of RNA metabolic processes” was chosen for the analysis. It was represented by genes that were observed to be downregulated during the in vitro maturation. The investigated ontology group includes 18 different genes such as androgen receptor (AR), inhibin beta A (INHBA), WW domain-containing transcription regulator 1 (WWTR1), oncogene FOS (FOS), mads box transcription enhancer factor 2 (MEF2C), vascular endothelial growth factor A (VEGFA), ikaros family zinc finger 2 (IKZF2), Indian hedgehog (IHH), rar-related orphan receptor A (RORA), mitogen-activated kinase kinase kinase 1 (MAP3K1), nuclear factor of activated cells 5 (NFAT5), actin-dependent regulator of chromatin subfamily A member 1 (SMARCA1), early growth response 1 (EGR1), early growth response 2 (EGR2), microphthalmia-associated transcription factor (MITF), mothers against decapentaplegic homolog 4 (SMAD4), amyloid beta A4 precursor protein (APP), and nuclear receptor subfamily 5 group A member 1 (NR5A1). We hypothesized that expression of these genes may be regulated by the process of mRNA storage, which is significantly associated with oocytes maturation* in vitro*.

## 2. Material and Methods

### 2.1. Animals

In this study, we used 45 pubertal crossbred Landrace gilts bred on commercial local farm with mean age 155 days (range 140–170 days) and mean weight 100 kg (95–120 kg). Housing conditions and feed were equal for the animals (dependent on age and reproductive status). Our study was approved by the Local Ethic Committee.

### 2.2. Collection of Porcine Ovaries and Cumulus-Oocyte-Complexes (COCs)

After recovering in slaughter, the ovaries and reproductive tracts were transported to the laboratory within 40 min at 38°C in 0.9% NaCl. The ovaries were kept in 5% fetal bovine serum solution (FBS; Sigma-Aldrich Co., St. Louis, MO, USA) in PBS before following IVM. Subsequently, COCs were recovered from single large follicles (>5 mm) by puncturing with a 5 ml syringe and 20-G needle and placed in a sterile Petri dish. Then COCs were washed three times in PBS supplemented with 36 *μ*g/ml pyruvate, 50 *μ*g/ml gentamycine, and 0.5 mg/ml BSA (Sigma-Aldrich, St. Louis, MO, USA), counted, and morphologically evaluated, using the scale suggested by Jackowska et al. [6], under an inverted microscope Zeiss, Axiovert 35 (Lübeck, Germany). For the subsequent steps of the study, only grade I COCs presenting homogeneous ooplasm and compact cumulus cells were collected.

### 2.3. Assessment of Oocyte Developmental Competence by Brilliant Cresyl Blue (BCB) Test

Collected porcine oocytes were subjected to two BCB tests. As a result of the first one 300 grade I BCB-positive (BCB^+^) oocytes were selected and split into two groups. The first one, described as “before IVM,” contained 150 oocytes (3 × *n* = 50) and was directly subjected to molecular analysis. The second one, described as “after IVM,” contained 150 oocytes (3 × *n* = 50) which were first* in vitro* matured, and then when graded as BCB^+^ they were exposed to subsequent molecular analysis. Microarray assay was performed in three biological replicates.

Oocytes selected for BCB staining test were washed two times in Dulbecco PBS (DPBS) (Sigma-Aldrich, St. Louis, MO) supplemented with 50 IU/ml penicillin, 50 *μ*g/ml streptomycin (Sigma-Aldrich, St. Louis, MO, USA), 0.4% [w/v] BSA, 0.34 mM pyruvate, and 5.5 mM glucose. Afterwards, oocytes were incubated for 90 min in 13 *μ*M BCB (Sigma-Aldrich, St. Louis, MO) in DPBS at 38.5°C and 5% CO_2_ humidified atmosphere. Subsequently, the oocytes were washed two times in DPBS and classified under an inverted microscope as stained blue (BCB^+^) or colourless (BCB^–^). In order to remove compact layers of cumulus cells, immature BCB^+^ COCs were incubated for 2 min with bovine testicular hyaluronidase (Sigma-Aldrich, St. Louis, MO, USA) at 38°C, then vortexed in 1% sodium citrate buffer, and mechanically displaced with a use of small-diameter glass micropipette. Granulosa-cell-free BCB^+^ oocytes were partially subjected to microarray assay (before IVM group) and partially to IVM followed by second BCB test and microarray assay (after IVM group).

### 2.4. *In Vitro* Maturation of Porcine Cumulus-Oocyte-Complexes (COCs)

The BCB^+^ COCs were matured in Nunclon™Δ 4-well dishes covered with mineral oil in 500 *μ*l standard porcine IVM culture medium, TCM-199 (tissue culture medium) with Earle's salts, and* L*-glutamine (Gibco BRL Life Technologies, Grand Island, NY, USA) supplemented with 0.1 mg/ml sodium pyruvate (Sigma-Aldrich, St. Louis, MO, USA), 2.2 mg/ml sodium bicarbonate (Nacalai Tesque, Inc., Kyoto, Japan), 0.1 mg/ml cysteine Sigma-Aldrich (St. Louis, MO, USA), 10 mg/ml BSA (bovine serum albumin) (Sigma-Aldrich, St. Louis, MO, USA), 10% (v/v) filtered porcine follicular fluid and gonadotropin supplements at final concentrations of 2.5 IU/ml hCG (Ayerst Laboratories, Inc., Philadelphia, PA, USA), and 2.5 IU/ml eCG (Intervet, Whitby, ON, Canada). The COCs were cultured at 38°C under 5% CO_2_ in humidified atmosphere for 22 h and then for additional 22 h in medium without hormones. Thereafter, the BCB staining test was performed once again and only BCB^+^ oocytes were taken for the following analysis.

### 2.5. RNA Extraction from Porcine Oocytes

For total RNA isolation, TRI reagent (Sigma, St. Louis, MO, USA) and RNeasy MinElute cleanup Kit (Qiagen, Hilden, Germany) were used. The concentration of total RNA was measured from the optical density at 260 nm, and the RNA purity was determined based on the 260/280 nm absorption ratio (higher than 1.8) (NanoDrop Spectrophotometer, Thermo Scientific, ALAB, Poland). Furthermore, the integrity and quality of RNA were obtained from the Bioanalyzer 2100 (Agilent Technologies, Inc., Santa Clara, CA, USA), resulting in RNA integrity numbers (RINs) ranging from 8.5 to 10 with an average of 9.2. Each sample of RNA was diluted to 100 ng/*μ*l with an OD260/OD280 ratio of 1.8/2.0, and for subsequent microarray assay, 500 ng of RNA from each sample was taken.

### 2.6. Microarray Expression Analysis and Statistics

The Affymetrix procedure and methods of analyzes were described previously [7–9]. cDNA was obtained from total RNA (100 ng) in the process of two round sense cDNA amplification (Ambion WT Expression Kit). Obtained cDNA was labeled and fragmented by Affymetrix GeneChip® WT Terminal Labeling and Hybridization (Affymetrix). Biotin-labeled fragments of cDNA (5.5 *μ*g) were hybridized to Affymetrix® Porcine Gene 1.1 ST Array Strip (48°C/20 h). Subsequent washing and staining were performed according to the technical protocol, using Affymetrix GeneAtlas Fluidics Station. The array strips were scanned employing Imaging Station of GeneAtlas System. The quality of gene expression data was checked according to quality control criteria provided by the Affymetrix GeneAtlasTM Operating Software. Downstream analysis was performed on the obtained CEL files.

The subsequent analyses were performed using BioConductor software, based on the statistical R programming language. Robust Multiarray Averaging (RMA) algorithm (“affy” package of BioConductor) was implemented for background correction, normalization, and summation of obtained CEL files. Obtained normalized dataset was merged with biological annotation taken form BioConductor “oligo” package. The significantly changed genes have been chosen based on *p* value breath 0.05 and expression fold higher than |2|. Statistical significance of changes in expression levels of analyzed genes was determined by moderated *t*-statistics from the empirical Bayes method. Obtained *p* value was corrected for multiple comparisons using the Benjamini and Hochberg's false discovery rate.

Differently expressed genes were subjected to the functional annotation clustering with DAVID (Database for Annotation, Visualization, and Integrated Discovery). Gene symbols from differently expressed genes were loaded to DAVID by “RDAVIDWebService” BioConductor package. Functional annotation charts generated by DAVID with overrepresented gene annotations were shown as bubble plots from BACA BioConductor package (https://cran.r-project.org/web/packages/BACA/BACA.pdf). Annotations from bubble plots have been chosen by following criteria: adjusted *p* value < 0.05, adjusted method = Benjamini, min number of genes per group = 5.

Interactions between proteins coded by genes and genes themselves from “positive regulation of RNA metabolic process” gene ontology group were investigated by STRING10 software (Search Tool for the Retrieval of Interacting Genes) [10]. STRING database contains information of protein/gene interactions, including experimental data, computational prediction methods, and public text collections. STRING database engine provided us with molecular interaction network formed between interested genes. Searching criteria are based on cooccurrences of genes/proteins in scientific texts (text mining), coexpression, and experimentally observed interactions. Besides interaction prediction, STRING also allows us to perform functional enrichments of GO terms based on previously uploaded gene set from “positive regulation of RNA metabolic process” GO BP term.

### 2.7. Gene Set Enrichment Analysis (GSEA)

GSEA is a computational method used for testing a priori defined gene sets (GO terms, pathways) for association with one of the two compared biological groups. The method uses Kolmogorov Smirnov (K-S) statistical test for identification of significantly enriched or depleted groups of genes [11]. The analysis was performed on GSEA Java Desktop Application from Broad Institute (http://software.broadinstitute.org/gsea/index.jsp). Normalized data from all of the genes from microarray were transformed to an appropriate format and imported to application. The predefined gene sets of genes have been selected from Molecular Signatures Database (MsigDB) [12]. Each gene belonging to the selected set was ranked according to the difference in their expression level using signal-to-noise ratio with 1000 times permutation. Based on that list of ranked genes, the enrichment score (ES) was calculated for each selected gene set. Enrichment score was calculated by sum statistic when a gene was present in the gene set and decreasing it when it was not [13]. Subsequently enrichment scores were normalized by their gene set size and false positive findings were corrected by FDR. The significant gene sets were considered to be those with adjusted *p* value < 0.05.

## 3. Results

**Figure 1:**
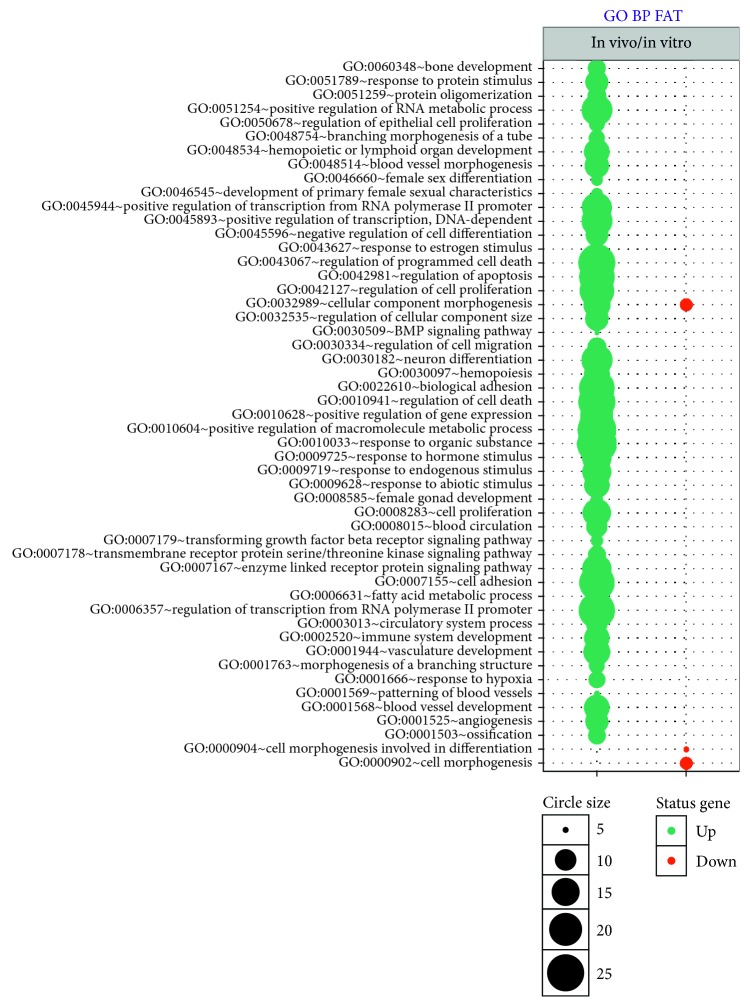
Bubble plot of genes annotations from DAVID GO BP database. Bubble plot of genes annotations from DAVID GO BP database wherein genes belonging to GO BP terms fulfill the following criteria: adjusted *p* value < 0.05, min number of genes > 5. The size of each bubble reflects the number of differentially expressed genes, assigned to the GO BP terms. Gene ontology terms with GO numbers are also shown. Downregulated genes forming selected GO terms were marked by turquoise colour.

**Figure 2:**
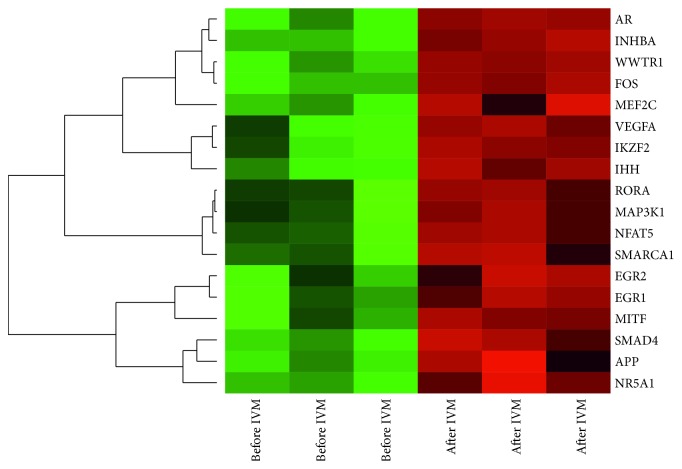
Heatmap representation of differentially expressed genes belonging to the “positive regulation of RNA metabolic process” functional category from DAVID GEOTERM BP database. Arbitrary signal intensity acquired from microarray analysis is represented by colours (green, higher; red, lower expression). log⁡2 signal intensity values for any single gene were resized to Row *Z*-Score scale (from −2, the lowest expression to +2, the highest expression for single gene).

Profiling of the whole transcriptome of the oocyte by Affymetrix microarray allowed us to analyze gene expression changes after* in vitro* maturation (after IVM) in relation to freshly isolated oocyte, before* in vitro* procedure (before IVM). By Affymetrix® Porcine Gene 1.1 ST Array, (available in GEO database accession: GSE97246), we examined expression of 12258 porcine transcripts (microarray data: supplementary materials). We considered genes fold change higher than |2| and with corrected *p* value lower than 0.05 they were considered as differentially expressed. This set of genes consists of 419 different transcripts. The first detailed analysis based on GO BP allowed us to identify 51 significantly enriched GO BP terms. Results of such analysis are presented as bubble plot in Figure 1. Some of the GO terms are in general level such as “response to organic substance,” whereas others are highly specific, for example, “regulation of epithelial cell proliferation.” Among of 51 different GO BP terms, three of them concern RNA metabolism involving “positive regulation of transcription from RNA polymerase II promoter,” “regulation of transcription from RNA polymerase II promoter,” and “positive regulation of RNA metabolic process.” One GO term described as “positive regulation of RNA metabolic process” was separated from other GO groups. The genes that belong to this term were subjected to hierarchical clusterization algorithm and presented as a heatmap graph (Figure 2).

The analyzed ontology group is formed by androgen receptor (AR), inhibin beta A (INHBA), WW domain-containing transcription regulator 1 (WWTR1), oncogene FOS (FOS), mads box transcription enhancer factor 2 (MEF2C), vascular endothelial growth factor A (VEGFA), ikaros family zinc finger 2 (IKZF2), Indian hedgehog (IHH), rar-related orphan receptor A (RORA), mitogen-activated kinase kinase kinase 1 (MAP3K1), nuclear factor of activated cells 5 (NFAT5), actin-dependent regulator of chromatin subfamily A member 1 (SMARCA1), early growth response 1 (EGR1), early growth response 2 (EGR2), microphthalmia-associated transcription factor (MITF), SMAD4, amyloid beta A4 precursor protein (APP), and nuclear receptor subfamily 5 group A member 1 (NR5A1). The symbols, names, fold changes, and corrected *p* values of differentially expressed genes belonging to “positive regulation of RNA metabolic process” are shown in Table 1. All of the presented genes were downregulated after* in vitro* maturation (fold change from −18.94 to −2.03). Among those genes, the strongest inhibitory effect to gene expression concerned the following: FOS gene (fold −18.94), VEGFA (fold −14.35), and AR (fold −9.44).

**Figure 3:**
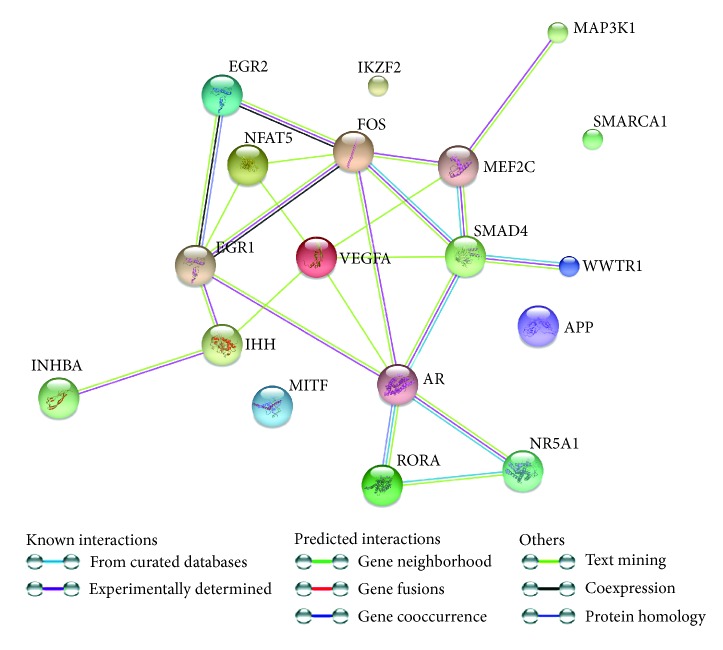
STRING-generated interaction network among differentially expressed genes belonging to the “positive regulation of RNA metabolic process” ontology group. The intensity of the edges reflects the strength of interaction score. Applied prediction methods: text mining, coexpression, and experimentally observed interactions.

Subsequently, we create STRING-generated interaction network on differently expressed genes from “positive regulation of RNA metabolic process” GO BP term. In the obtained results, the strongest interactions were observed between AR, VEGFA, MEF2C, RORA, N5A1, SMAD4, and FOS. The higher number of formed edges concerned SMAD4 and AR gene/protein (Figure 3).

One gene can belong to many GO terms. By this reason, we perform functional enrichments of GO terms based on previously uploaded gene set. Top five GO terms, from each of the main GO categories, that also contain genes belonging to “positive regulation of RNA metabolic process” GO BP were shown in Table 2.

**Figure 4:**
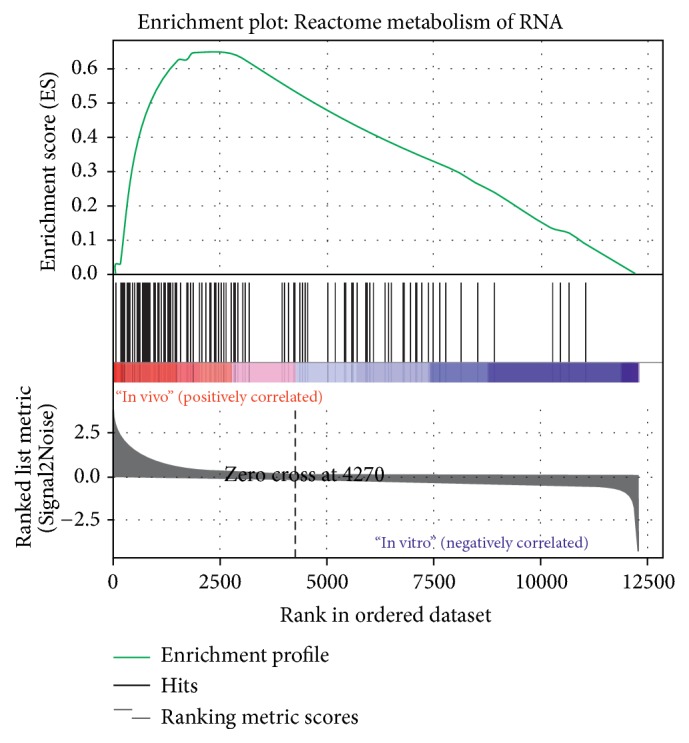
Enrichment plot of genes forming REACTOME_METABOLISM_OF_RNA term. Profile of the running enrichment score and positions of gene set members are mark on the rank ordered list.

Finally, the gene set enrichment analysis between oocyte genes before and after* in vitro* maturation revealed highly enrichment of genes belonging to “metabolism of RNA” gene set in oocytes before* in vitro* maturation. The obtained results might be also interpreted as downregulation of genes associated with RNA metabolism and regulation of gene expression, caused by oocyte* in vitro* maturation (Figure 4).

## 4. Discussion

The proper mammalian cumulus-oocytes complexes (COCs) maturation both* in vivo* and/or* in vitro* requires compound process of cell to cell-specific shuttling between female gamete and surrounding somatic cells. This bidirectional communication allows transporting small substances via paracrine system between these two populations of cells. It was well recognized that full maturation stage of oocytes is divided into two phases such as nuclear and cytoplasmic [14, 15]. The nuclear maturation involved several modifications of nuclear chromatin that finally leads to reaching female gamete MII stage. On the other hand, the accumulation in cell cytoplasm and large amount of mRNAs as a template for further protein synthesis is defined as cytoplasmic maturation. It was focused on several species of mammals that both nuclear and cytoplasmic maturation is necessary for formation of fully fertilizable oocyte, formation of zygotes, and proper embryos growth in preimplantation stage [16, 17]. Additionally, it was also observed that oocytes, which are unable to resume meiosis, cannot be fertilized by spermatozoon [18, 19]. Therefore, it is suggested that proper regulation of processes at early stages of oogenesis is required for complete maturation of oocytes. Moreover, there are still large differences in efficiency of maturation between* in vivo* and* in vitro* conditions, and therefore researches based on focusing of molecular markers of this process are right now of high interest.

In our study, employing a microarray approach, we aimed to investigate the transcriptome profile of porcine oocyte before (before IVM group) and after (after IVM group) 44 hours of* in vitro* maturation (IVM). The obtained results indicate the decrease in mRNA expression of genes associated with RNA metabolism and regulation of gene expression is caused by oocyte* in vitro* maturation. From all of 18 presented, downregulated genes belonging to “positive regulation of RNA metabolic process” GO BP term category, the strongest inhibitory effect to gene expression concerned a transcription factor FBJ murine osteosarcoma viral oncogene homolog (FOS). FOS is a protooncogene that is a member of the immediate-early response family of transcription factors. This family has been implicated as regulators of cell proliferation, differentiation, and transformation. FOS is a transcription factor which can heterodimerize with members of the Jun family to form the transcription factor activator protein 1 (AP-1) [20]. Original literature sources indicate presence of FOS transcripts in the germinal vesicle oocytes and meiosis II stage oocytes [21, 22].

In the present study, we observed decreased mRNA expression also for other transcripts classified, like FOS, into cellular growth and proliferation (VEGFA, EGR1, and EGR2) functional group. Interestingly, vascular endothelial growth factor A (VEGFA) showed the second strongest, after FOS, inhibitory effect to gene expression (fold −14.35), while EGR2 showed the fourth largest change (fold −6,04) after* in vitro* maturation. These data point to a key role of genes belonging to this functional group as the factors affecting* in vivo* regulation of RNA metabolic process. VEGFA is well established as a critical regulator of angiogenesis in the ovulatory follicle [23]. Moreover, in addition to mediation of endothelial cells angiogenic process, studies with human ovarian microvascular endothelial cells (hOMECs) prove that VEGFA promote endothelial cell migration [24]. A study by Li et al. indicates improved quality and survival rate of subcutaneously transplanted mouse ovarian tissue in VEGF coupled with FGF2-treated subjects [25]. Furthermore,* in vitro* maturation of bovine oocytes supplemented with VEGF results in an improvement of cytoplasmic maturation, with a positive impact on oocyte developmental capacity [26]. Early growth response (EGR) family members are highly homologous and contain conserved DNA-binding domain composed of three zinc-finger motifs [27]. These transcription factors recognize a nine-base-pair segment of DNA in promoter of target genes and then participate in regulating their expression. Presence of the domain of interaction with the transcriptional corepressors NGFI-A-1/2 suggests that EGR1, EGR2, and EGR3 (but not EGR4) can lead to transcriptional repression [28]. Additionally, EGR2 (together with EGR3) is required for efficient AP-1 transcription factor activation of the in B and T cells after antigen receptor signaling [29]. This study indicates decreased mRNA expression of two repressors from this family: EGR1 and EGR2. Our results may suggest greater importance of EGR2 for proper oocyte maturation and EGR2 showed clearly stronger inhibitory effect on gene expression compared with EGR1. To confirm this request, studies with knockout mice indicated that EGR1 is not critically involved in prenatal development, because EGR2 knockout is lethal, whereas mice lacking EGR1 are viable despite reduced body size, sterility associated with alterations of the pituitary-gonadal axis, as well as axial myopia [30, 31]. Nevertheless, EGR1 is functionally implicated in numerous critical biological processes, including inflammation, cell proliferation, differentiation, vascular wound response, and cancer progression [32].

Data from Geh et al. 2011 studies suggest that c-Jun (together with FOS part of the transcription factor activator protein 1 (AP-1)) regulates mitogen-activated protein kinase kinase kinase 1 (MAP3K1) promoter, a member of the MAP3K superfamily. This member of intracellular signaling kinases also showed decreased mRNA expression after* in vitro* maturation in our study. On the one hand, AP-1, via c-Jun, regulates MAP3K1 expression, and on the other hand, kinase activity of MAP3K1 is required for c-Jun phosphorylation and maximal AP-1 activity [33]. In relation to early stages of fetal development, one of the most obvious functions of MAP3K1 is the control of eyelid closure [34]. In addition, eyelid closure requires, except the participation of MAP3K1, presence of nuclear transcription factors, like SMAD. SMAD family member 4 (SMAD4), belonging to the SMAD proteins family, is known to be a central mediator of transforming growth factor beta (TGF-*β*) signaling pathway. SMAD4 is an intracellular signal transducer that acts downstream of the receptors for TGF-*β* family members. These molecules, after receiving a signal from activated TGF-*β*, activate target gene transcription in association with DNA-binding partners [35]. It is worth emphasizing that, in addition to the typical TGF-*β* signaling pathway generated by the SMAD family, there are also pathways independent of the activity of these proteins. Regarding our result, it should be noted that the group of factors that are an alternative to SMAD pathway includes MAP3K1.

Androgen receptor (AR) signaling pathway plays a critical role in reproductive function in both males and females. Many studies demonstrate that androgen signaling contributes to normal follicle development, ovulation, and fertility [36]. AR expression in all mammalian ovaries clearly supports a universal role for AR-mediated androgen actions in influencing ovarian follicle development. Furthermore, AR is expressed throughout most stages of follicular development. Even in pig fetal ovaries, expression of these proteins has been detected [37]. Furthermore, AR immunostaining is observed in the oocyte, granulosa cells, and theca cells of rat preantral follicles [38]. In summarizing, AR is expressed in each of the 3 types of cells in the ovary: theca interstitial cells where androgens are produced, granulosa cells where testosterone is converted to estrogen, and the germ cells, the oocytes [39]. Abreu-Martin laboratory presented results suggesting that the MAP3K1 pathway plays a role in modulating the transcriptional response of the androgen receptor to ligands [40]. Studies employing global AR knockout (ARKO) female mice [41, 42], according to well-defined roles of AR in male reproduction, indicate that these subjects are subfertile, have defective folliculogenesis, and ultimately develop premature ovarian failure. Furthermore, for even more precise definition where androgens are acting in the hypothalamic-pituitary-gonadal axis to regulate follicle growth and female fertility, female mice with AR knocked out in granulosa cells (GCARKO) [43, 44] and with theca-specific deletion of AR (ThARKO) [45] were created. GC-specific ARKO female mice have reduced fertility and altered estrous cycling, while Ma laboratory showed that AR expression in theca cells likely does not influence fertility nor androgen levels in female mice. Our results indicate lower AR's transcript levels in porcine oocyte after IVM. To this day, one oocyte cell-specific ARKO (OoARKO) has been created. OoARKO females were reported to have normal fertility, oestrous cycles, follicle populations, and CL numbers [43]. However, oocyte maturation (GVBD) induced* in vitro* by a high concentration of the nonaromatizable androgen, DHT, was significantly reduced in OoARKO oocytes. These findings imply that while AR oocyte actions are not essential for overall ovarian function and female fertility, they may play an important role when intraovarian levels of androgens are elevated. Additionally, we may postulate that androgen receptor signaling pathway plays important role in oocyte maturation.

## Figures and Tables

**Table 1:** Fold changes, adjusted *p* values of differentially expressed genes.

Gene symbol	Gene name	Fold change	Adj. *p* value
FOS	FBJ murine osteosarcoma viral oncogene homolog	−18.94	0.00005
VEGFA	Vascular endothelial growth factor A	−14.35	0.00191
AR	Androgen receptor	− 9.44	0.00014
EGR2	Early growth response 2	− 6.04	0.00795
INHBA	Inhibin, beta A	− 4.14	0.00015
IHH	Indian hedgehog	− 3.28	0.00055
APP	Amyloid beta (A4) precursor protein	− 3.09	0.00560
WWTR1	WW domain containing transcription regulator 1	− 3.06	0.00025
SMARCA1	SWI/SNF related, matrix associated, actin dependent regulator of chromatin, subfamily a, member 1	− 3.04	0.01476
NFAT5	Nuclear factor of activated T-cells 5, tonicity-responsive	− 2.90	0.01315
SMAD4	SMAD family member 4	− 2.72	0.00124
MAP3K1	Mitogen-activated protein kinase kinase kinase 1, E3 ubiquitin protein ligase	− 2.71	0.02475
EGR1	Early growth response 1	− 2.66	0.00548
RORA	RAR-related orphan receptor A	− 2.60	0.02155
NR5A1	Nuclear receptor subfamily 5, group A, member 1	− 2.35	0.00189
IKZF2	IKAROS family zinc finger 2 (Helios)	− 2.32	0.00298
MEF2C	Myocyte enhancer factor 2C	− 2.20	0.00396
MITF	Microphthalmia-associated transcription factor	− 2.03	0.00633

Fold changes and adjusted *p* values of differentially expressed genes belonging to the “positive regulation of RNA metabolic process” functional category from DAVID GEOTERM BP database. Symbols and names of the selected genes are also shown.

**Table 2:** Top five GO categories formed by differentially expressed genes.

Pathway ID	Pathway description	Count in gene set	False discovery rate
Biological process (GO)			
GO:0042221	Response to chemical	8	3.42*e* − 09
GO:0051716	Cellular response to stimulus	9	3.42*e* − 09
GO:0016070	RNA metabolic process	7	3.62*e* − 09
GO:0007154	Cell communication	8	4.56*e* − 09
GO:0007165	Signal transduction	8	4.56*e* − 09

Molecular function (GO)			
GO:0043565	Sequence-specific DNA binding	6	1.05*e* − 09
GO:0000981	RNA polymerase II transcription factor activity, sequence-specific DNA binding	5	2.94*e* − 09
GO:0001077	Transcriptional activator activity, RNA polymerase II core promoter proximal region sequence-specific binding	4	4.59*e* − 08
GO:0003700	Transcription factor activity, sequence-specific DNA binding	5	4.59*e* − 08
GO:0000977	RNA polymerase II regulatory region sequence-specific DNA binding	4	2.17*e* − 07

Cellular component (GO)			
GO:0005575	Cellular component	9	1.09*e* − 05
GO:0005634	Nucleus	6	1.09*e* − 05
GO:0005623	Cell	8	1.77*e* − 05
GO:0043231	Intracellular membrane-bounded organelle	7	1.77*e* − 05
GO:0031981	nuclear lumen	4	5.15*e* − 05

Top five GO categories formed by genes differentially expressed belonging to the “positive regulation of RNA metabolic process” ontology group. GO categories were generated in STRING software. GO ID (pathway ID), GO term description (pathway description), and number of the genes belonging to appropriate category (count in gene set) are shown.

## Supplementary Materials

Supplementary Materials The MIME (Minimal Information about a Microarray Experiment) of porcine oocytes maturation* in vitro* described in GEO (Gene Expression Omnibus).
